# Assessment of plan–execution fidelity in a fully digital workflow using custom-made plates for mandibular fracture repair: a prospective pilot study

**DOI:** 10.1186/s12903-026-09349-5

**Published:** 2026-07-22

**Authors:** Riad Kamal Riad, Eman Shalaby, Hesham ElHawary

**Affiliations:** Department of Oral and Maxillofacial Surgery, Faculty of Dentistry, Cairo University, Cairo, Egypt

**Keywords:** Mandibular fractures, Fracture fixation, Internal, Surgery, Computer-assisted, Printing, Three-dimensional, Patient-specific modeling, Imaging, Three-dimensional, Quality of life, Plan–execution fidelity

## Abstract

**Background:**

Virtual surgical planning (VSP) and custom-made plates (patient-specific implants, PSIs) are increasingly used in mandibular fracture repair, but quantitative data on plan–execution fidelity remain limited. Plan–execution fidelity refers to the geometric agreement between the virtually planned and postoperative mandibular morphology after image registration. The aim of this pilot study was to quantify plan–execution fidelity and to describe patient-reported recovery in a fully digital, custom-plate workflow for non-comminuted mandibular fractures, while generating hypotheses about plate design.

**Methods:**

Six patients with non-comminuted mandibular body or parasymphyseal fractures underwent open reduction and internal fixation (ORIF) using a fully digital workflow. Plan–execution fidelity (the primary feasibility outcome) was assessed as the mean absolute error (MAE) of 10 predefined measurements after iterative closest point (ICP) registration on the contralateral ramus and condyle (condylar metrics excluded a priori). Measurements were performed by three blinded observers. The Oral Health Impact Profile-14 (OHIP-14) was recorded preoperatively and at 1, 4, and 12 weeks. Analyses are purely descriptive; no inferential statistics are reported.

**Results:**

In this six-patient cohort, the primary (patient-level) MAE ranged from 0.25 to 0.81 mm (mean 0.48 mm, SD 0.21 mm). At the level of individual measurements (60 readings from the same six patients), the MAE was 0.45 mm. OHIP-14 total scores improved from 48.2 (SD 5.6) preoperatively to 5.2 (SD 4.1) at 12 weeks. Plate design, indication, and chronological order were structurally confounded; therefore, no comparative interpretation is possible.

**Conclusions:**

In this pilot cohort, a fully digital workflow using custom-made plates was associated with submillimetre plan–execution deviation (mean 0.48 mm) within the registration framework used. The workflow was technically feasible and showed close correspondence between the virtual plan and postoperative morphology, although this geometric agreement should not be interpreted as independent anatomical accuracy. These preliminary findings provide a basis for future controlled studies aimed at validating clinical relevance, workflow efficiency, and cost-effectiveness in larger patient cohorts.

**Trial registration:**

ClinicalTrials.gov NCT07499726; registered 24 March 2026 (2026-03-24), retrospectively, after initiation of recruitment.

**Supplementary Information:**

The online version contains supplementary material available at 10.1186/s12903-026-09349-5.

## Introduction

Mandibular fractures are the most common facial bone injuries, accounting for 36–70% of all maxillofacial fractures [1]. Open reduction and internal fixation (ORIF) remains the standard of care, aiming to restore anatomical alignment and occlusion. Conventional fixation relies on intraoperative plate adaptation, which is operator dependent, may introduce inaccuracies, and can be time consuming [2, 3]. Digital workflows incorporating virtual surgical planning (VSP), intraoral scanning, and custom-made plates (patient-specific implants, PSIs) offer potential advantages, including preoperative plate design and elimination of intraoperative bending [4–6]; their application to mandibular fracture repair has recently been the subject of systematic review [7].

However, their application in routine mandibular trauma remains insufficiently studied — particularly the quantitative assessment of plan–execution fidelity. Plan–execution fidelity refers to the geometric agreement between the virtually planned and postoperative mandibular morphology after image registration. Importantly, plan–execution fidelity reflects agreement between the planned and achieved result and should not be interpreted as independent validation of anatomical accuracy [8]. Earlier reports of patient-specific implants in mandibular trauma have addressed atrophic, edentulous, or angle fractures [9, 10], but have not quantified how faithfully a fully digital, occlusion-driven plan is transferred to the patient in dentate body and parasymphyseal fractures.

The workflow evaluated here rests on a specific premise: the virtual reduction is not an arbitrary digital construct but is performed according to the same established principles that govern conventional mandibular fracture reduction — restoration of a correct occlusal relationship, which is the reason a preoperative intraoral scan of both dental arches is incorporated, together with re-establishment of bony continuity across the fracture, judged by realignment of the buccal and lingual cortical plates and of the inferior mandibular border [11–13]. A patient-specific plate is then designed solely to hold the segments in this reduced position. Consequently, the clinically relevant question is not whether the digital plan is internally consistent, but whether that principled plan can be transferred to the patient with sufficient fidelity — that is, whether virtual planning is a reliable basis on which to plan treatment. Establishing this transfer reliability is a necessary precondition for any subsequent comparative trial: under staged frameworks for the evaluation of surgical innovation, early feasibility and development studies that characterise a new technique and its reproducibility logically precede randomised comparison [14]. The present study was therefore deliberately designed as a single-arm feasibility evaluation without a control group; a control arm would be premature before the reliability of plan transfer has been demonstrated and would not address the question posed here. If the workflow proves reliable, it can then justifiably proceed to randomised controlled trials and the development of a standardised treatment protocol.

Accordingly, this prospective single-arm pilot study set out to determine whether the fully digital workflow — virtual surgical planning, an occlusal stent, a surgical guide, and a patient-specific plate — could be executed such that the virtually planned reduction was clinically achieved, with clinical achievement judged against the two accepted reference standards for mandibular fracture treatment: a stable, functional occlusion and re-established bony continuity with uneventful union [13, 15]. The study aimed to (1) quantify plan–execution fidelity as a geometric measure of how faithfully the executed result reproduced the digital plan; (2) describe patient-reported outcomes using OHIP-14; and (3) generate hypotheses regarding plate-design features, while acknowledging that design-related comparisons are structurally confounded and cannot support causal inference.

## Materials and methods

### Study design

Prospective, single-arm pilot study conducted at the Department of Oral and Maxillofacial Surgery, Cairo University. Approved by the Ethics Committee of the Faculty of Dentistry, Cairo University (Approval No. 22624/2023). All procedures complied with the Declaration of Helsinki. The study was registered retrospectively after initiation of patient recruitment; no changes to outcome measures or analysis plan occurred after registration. Written informed consent was obtained from all participants.

### Participants

Six adult patients (18–65 years) with isolated, displaced mandibular body or parasymphyseal fractures requiring ORIF were included. The cohort was deliberately restricted to a single, homogeneous fracture type. Comminuted, pathological, panfacial, and multi-region fractures were excluded so as to unify the fracture pattern and avoid introducing additional variables — such as fragment number, segmental bone loss, and concomitant midface injury — that are known to alter reduction demands, fixation strategy, and outcomes, and that would otherwise confound a pilot evaluation of plan–execution fidelity [16].

#### Inclusion criteria

Isolated, displaced fracture of mandibular body or parasymphysis; age 18–65 years; sufficient dentition (≥ 10 natural or restored teeth per arch) to allow stable seating of the occlusal stent (threshold selected pragmatically).

#### Exclusion criteria

Comminuted fractures, pathological fractures, concomitant midface fractures, uncontrolled systemic disease, pregnancy, absence of a postoperative CT scan (refusal or medical contraindication).

#### Sample size justification

No formal power calculation was performed. This was conceived as a preliminary, first-stage pilot study whose purpose was to establish the technical feasibility of the fully digital workflow and to generate descriptive parameter estimates (such as the magnitude and dispersion of plan–execution deviation) to inform the design of future adequately powered studies, rather than to test a hypothesis. Accordingly, the sample size (*n* = 6) was selected pragmatically on the basis of feasibility, resource availability, and established guidance for pilot and feasibility studies, which recommends that such samples be determined by practical considerations rather than by formal power calculations and that modest sample sizes are appropriate for this exploratory purpose [17, 18]. The cohort was further confined to a single homogeneous fracture type (isolated, non-comminuted body or parasymphyseal fractures) to unify the fracture pattern and minimise the influence of factors — comminution, segmental defects, concomitant facial fractures, and multi-region involvement — that are recognised to affect reduction accuracy and clinical outcome and that would otherwise confound the fidelity estimate in a small cohort [16]. The study does not define a minimum detectable error difference; therefore, the sample size is justified solely for descriptive pilot objectives.

### Preoperative imaging and digital workflow

All patients underwent preoperative high-resolution computed tomography (CT) (slice thickness 0.625 mm, 120 kVp, 80 mAs, field of view 180 mm, 64-slice scanner, Siemens Somatom Definition AS). Digital Imaging and Communications in Medicine (DICOM) data were imported into Mimics Medical (version 24.0, Materialise NV). Concurrently, intraoral digital impressions of both arches were obtained using a Runyes 3DS V5 scanner (stated laboratory accuracy ± 15 μm). Scans were exported as standard tessellation language (STL) files.

### Virtual fracture reduction

**Fig. 1:**
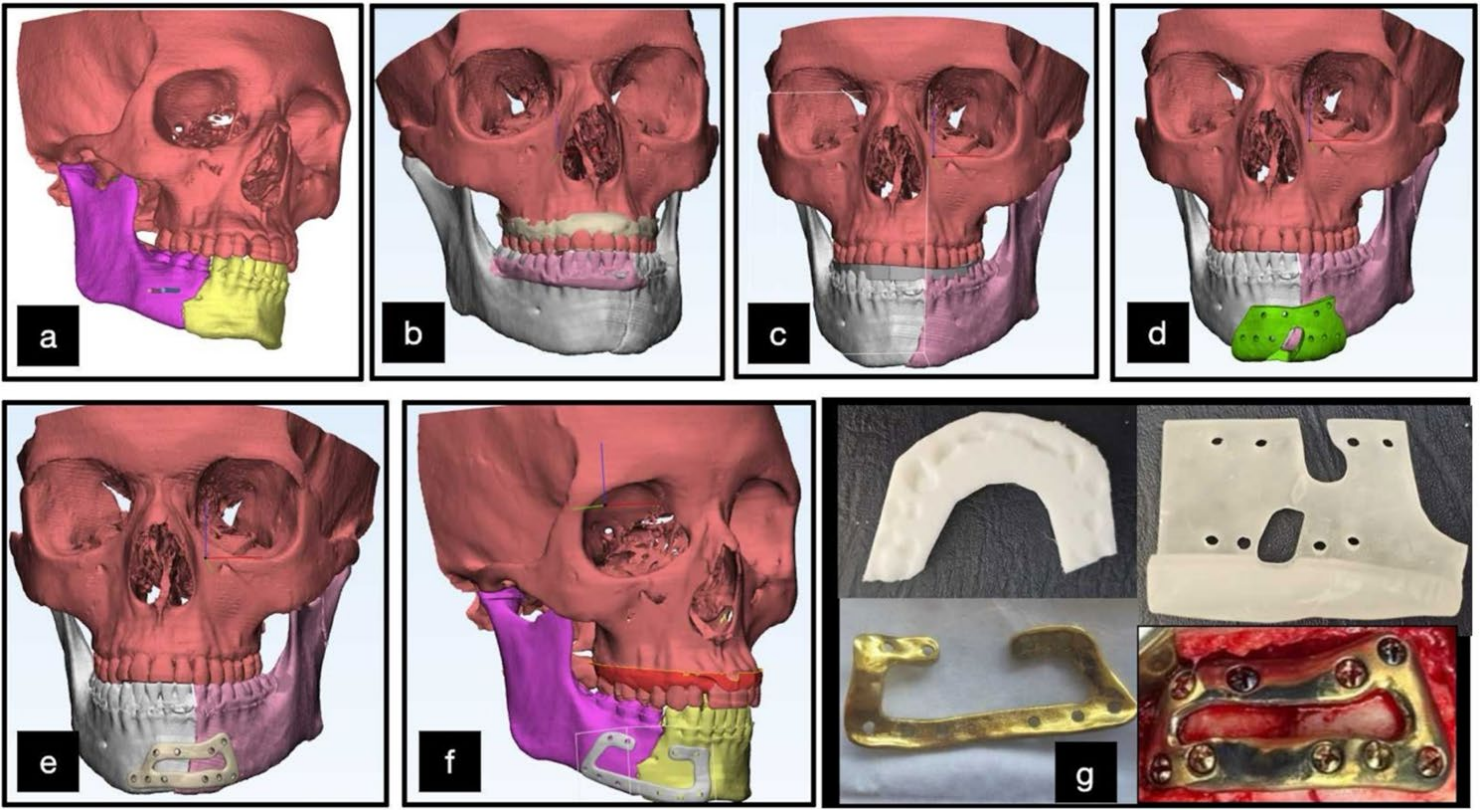
Virtual planning and custom component design. **a** Segmentation of the mandible from preoperative CT (threshold 226–3071 HU). **b** Virtual fracture reduction with occlusal verification; green indicates acceptable contact (< 0.1 mm, pragmatically defined). **c** 3D printed occlusal stent spanning both dental arches. **d** Surgical guide with cylindrical drill sleeves (inner diameter 1.8 mm). **e** Open superior PSI design (gap in superior component to bypass mental nerve) — used in Cases 1–2. **f** Closed PSI design (continuous box construct) — used in Cases 3–6. **g** The printed occlusal stent, surgical guide, and the milled PSIs

Virtual reduction was performed to satisfy the established goals of mandibular fracture reduction — restoration of the occlusal relationship and re-establishment of bony continuity across the fracture — using the intraoral scan to define occlusion and the contralateral mandible as a morphological reference. CT data were thresholded (226–3071 Hounsfield units, HU). The mandible was segmented, then re-segmented at the fracture site (Fig. 1a, b). Intraoral scans were superimposed onto CT bone models using iterative closest point (ICP) registration. The contralateral ramus and condyle were manually isolated from the virtual reduction model by removing the ipsilateral fracture segments; this region was then used as the reference. ICP was performed using a point-to-plane algorithm under standard software convergence settings, with convergence defined as stabilisation of root-mean-square (RMS) change < 0.01 mm between iterations. Registration was restricted to the contralateral ramus and condyle (the most stable available reference; cranial base registration not feasible due to limited CT field of view).

Internal plausibility of the virtual reduction was supported by restoration of mandibular continuity, visually symmetric gonial contours, and absence of segmental overlap or step deformities on three-dimensional (3D) inspection. Reduction was accepted only when all three established reduction criteria were satisfied: (1) continuity of the inferior mandibular border, (2) alignment of the buccal and lingual cortices, and (3) restoration of the occlusal relationship (virtual intercuspal position). Occlusal contacts were required to be within 0.1 mm at three or more points per quadrant, as determined within the planning software environment — a pragmatically defined threshold based on clinical feasibility of stent fit.

### Design of custom-made components

**Fig. 2:**
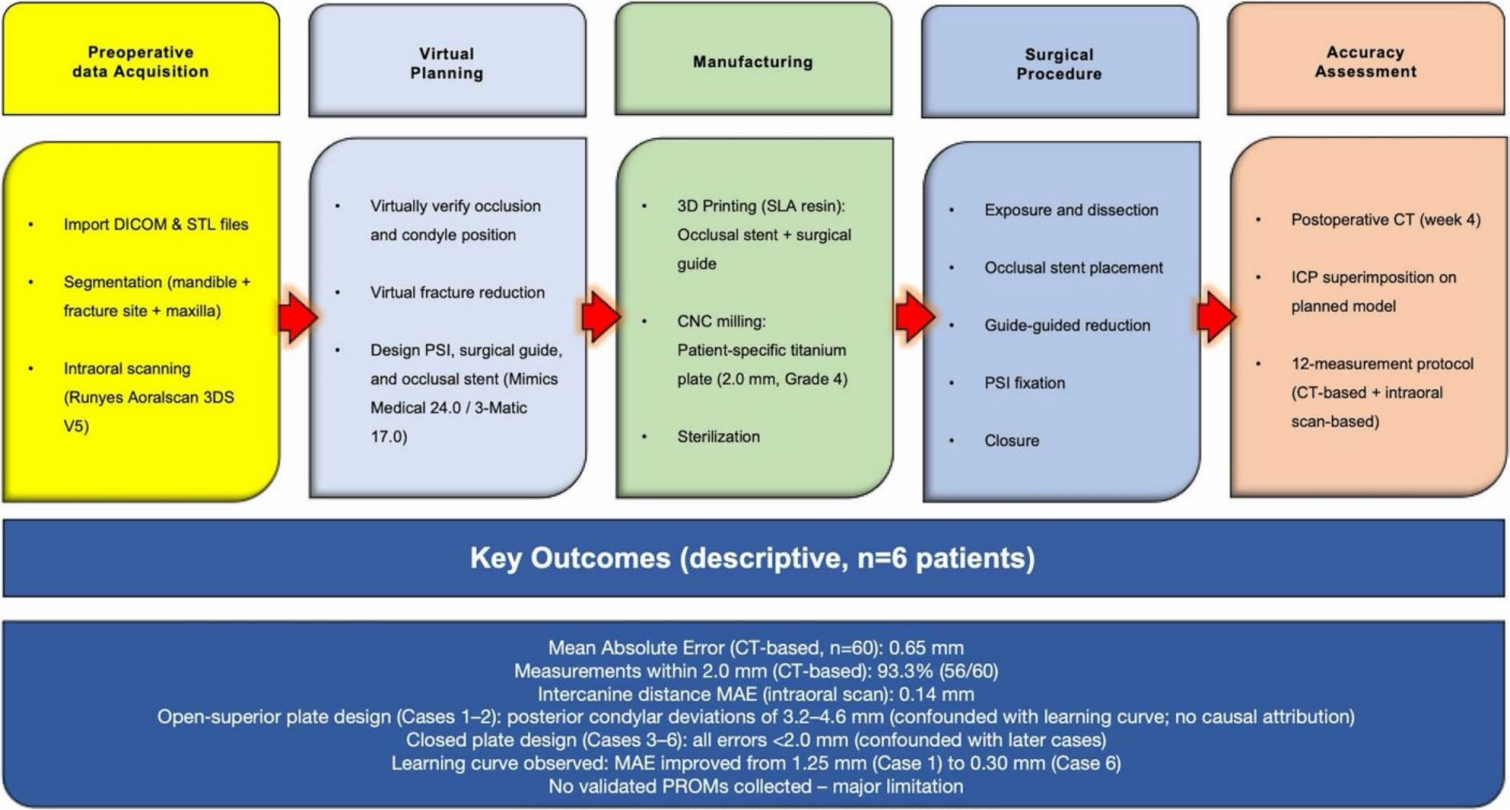
Complete digital workflow schematic. Flowchart illustrating the sequence: preoperative CT and intraoral scanning → virtual fracture reduction (verified by inferior border continuity, cortical alignment, and occlusion) → design of occlusal stent, surgical guide, and PSI → 3D printing and CNC milling → intraoperative placement → postoperative CT and agreement assessment

Three custom components were designed using Mimics Medical + 3-Matic v17.0: (1) an occlusal stent spanning both arches, secured with circumdental wiring; (2) a surgical guide with cylindrical sleeves (inner diameter 1.8 mm); and (3) a patient-specific titanium plate (Grade 4, 2.0 mm thick). Two plate designs were used: an open superior design (Cases 1–2) with a gap in the superior component to bypass the mental nerve and two vertical bridging arms (each 3.0 mm wide, 2.0 mm thick); and a closed design (Cases 3–6) with a continuous box construct (Fig. 1c–g). The complete digital workflow, from imaging to postoperative assessment, is summarised in Fig. 2.

### Manufacturing

Occlusal stents and surgical guides were 3D printed using stereolithography (Form 3B, Dental SG Resin, layer thickness 50 μm). Custom-made plates were computer numerical control (CNC) milled (Material Technology Innovations, Cairo; tolerance ± 0.05 mm), heat treated, polished, cleaned, and sterilised. Specific data on individual planning versus manufacturing times were not prospectively recorded; however, the total interval from CT to surgery did not exceed 7 days in any case.

### Surgical procedure

**Fig. 3:**
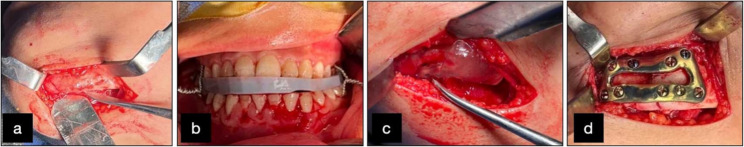
Intraoperative surgical steps. **a** Consistent submandibular approach with exposure of fracture. **b** Placement and circumdental wiring of the 3D printed occlusal stent to obtain intermaxillary fixation. **c** Positioning of the patient-specific surgical guide on the mandibular surface. **d** Securing the custom titanium plate with 2.0 mm self-tapping screws

All procedures were performed under general anaesthesia by a single experienced surgeon (> 28 years of experience) using a consistent submandibular approach. The occlusal stent was placed and secured with circumdental wires. The surgical guide was positioned, pilot holes drilled through the sleeves, and the custom plate fixed with 2.0 mm self-tapping screws (5 mm superior, 9 mm inferior). The stent was then removed, and occlusion and mandibular movements were checked (Fig. 3a–d). The study was not designed to evaluate workflow efficiency or potential treatment delay attributable to PSI fabrication; however, all surgeries were performed within 7 days of injury.

### Outcome measures

Two complementary categories of outcome were assessed. First, clinical achievement of the planned reduction — whether the fully digital plan was realised in the patient — was verified against the accepted clinical reference standards for mandibular fracture treatment, namely restoration of a stable, functional occlusion (assessed intraoperatively and at each follow-up visit) and re-establishment of mandibular bony continuity with uneventful union (assessed clinically and on the postoperative CT) [13, 15]; these clinical criteria, rather than comparison with the contralateral side or a separate control group, served to confirm that the digital steps were clinically achieved. Second, the geometric plan–execution fidelity (below) was quantified to describe how faithfully the executed result reproduced the digital plan.

#### Primary feasibility (Technical) outcome: plan–execution fidelity

##### Definition

Plan–execution fidelity is the registration-dependent geometric deviation between planned and postoperative mandibular morphology, quantified as the mean absolute error (MAE) of 10 predefined linear and angular measurements (Supplementary Table S1) after ICP registration on the contralateral ramus and condyle. This is a closed-loop digital congruence metric; it does not represent independent anatomical validation, and no validated threshold exists linking such fidelity measurements to clinical outcomes in mandibular fracture surgery. Accordingly, it serves as the primary feasibility (technical) outcome of this study rather than a patient-important clinical endpoint

##### Measurement acquisition

A postoperative CT scan was obtained at week 4 using the same parameters. Preoperative, planned, and postoperative mandibular models were aligned using ICP on stable segments (contralateral ramus and condyle). Acceptable registration was defined as RMS surface deviation < 0.5 mm (achieved mean 0.27 mm). Because ICP registration minimises geometric discrepancy on the selected reference segment, the measured deviations are not fully independent of the registration procedure itself. No routine radiographic follow-up beyond the 4-week CT was performed.

##### Measurement protocol

**Fig. 4:**
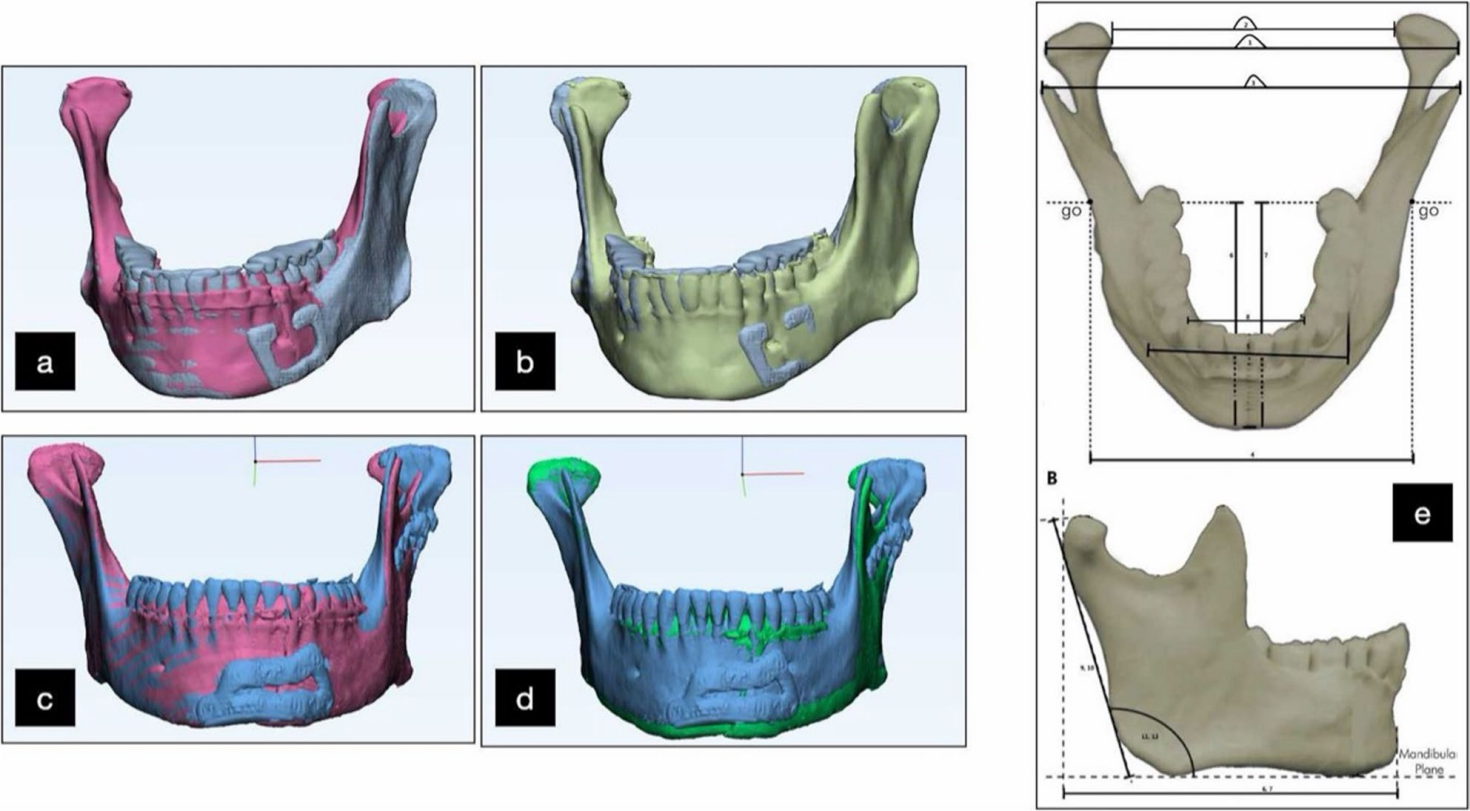
Superimposition and measurement protocol. **a**–**d** Superimposition of preoperative (blue), planned (green), and postoperative (red) mandibular models using stable reference segments (contralateral ramus and condyle). Registration restricted to these regions to minimise circularity. Cranial base registration was not feasible due to limited CT field of view. ICP convergence was defined by stabilisation of RMS change (< 0.01 mm between iterations), not by anatomical residual error. RMS registration error < 0.5 mm (achieved mean 0.27 mm). **e** Twelve standardised mandibular landmarks and measurements (see Supplementary Table S1 for definitions). CT-based measurements (1–7, 9–12) and intraoral-scan-based intercanine distance (8) are shown. Note: Measurements #1 and #2 (condylar) were designated exploratory and excluded from the primary plan–execution fidelity analysis due to their high sensitivity to registration reference instability

Twelve standardised mandibular measurements (linear and angular) were performed based on published protocols [19, 20] (Fig. 4). Condylar-based measurements (#1 lateral intercondylar length, #2 medial intercondylar length) were excluded from the primary composite a priori because they exhibit high sensitivity to registration reference instability. Primary analysis focused on the remaining 10 measurements (#3–12). Condylar metrics were reported separately as an exploratory composite.

##### Observer methodology and reliability

Observers underwent calibration on two non-study datasets before formal measurements were initiated. Measurements were then performed independently by three PhD-level oral and maxillofacial surgery trainees, each twice, one week apart, blinded to planned values. The mean of six readings per parameter was used. Reliability was assessed using a two-way random effects model with absolute agreement. Intra-observer reliability was quantified using the intraclass correlation coefficient (ICC), reported as ICC(2,1), and inter-observer reliability using ICC(2,k), according to the Shrout and Fleiss classification [21, 22]. Intra-observer ICC(2,1) ranged 0.92–0.94; inter-observer ICC(2,k) was 0.92. These values are sample specific (derived from six patients) and should be interpreted cautiously because reliability estimates from a very small sample may be unstable.

##### Summary metrics

Patient-level MAE (averaging 10 measurements per patient) is the primary feasibility (technical) summary metric. Measurement-level MAE (all 60 measurements) is reported secondarily. Standard deviations are provided as descriptive dispersion measures, not for inferential testing.

#### Patient-reported outcomes

The Arabic validated version of the Oral Health Impact Profile-14 (OHIP-14) was administered preoperatively and at 1, 4, and 12 weeks postoperatively [23, 24].

### Statistical approach and outcome hierarchy

All estimates are descriptive summaries of this dataset only. No inferential statistics (p values, confidence intervals, hypothesis tests) are reported. Confidence intervals are omitted due to instability with *n* = 6. Standard deviations are reported solely as within-sample dispersion. In addition to the mean, the median patient-level MAE is reported to account for possible skew in a small sample. Readers are cautioned that all numerical estimates are derived from only six independent patients; the reported precision (e.g., 0.48 mm) should not be misinterpreted as indicating high measurement certainty.

The outcome hierarchy is as follows:

- Primary feasibility (technical) outcome: Patient-level MAE (10-measurement composite, condylar metrics excluded).
- Secondary outcome: Measurement-level MAE (all 60 individual measurements).
- Exploratory outcomes: Condylar composite (#1 and #2) and OHIP-14 scores.

The primary endpoint is therefore a feasibility (technical) performance metric, consistent with the early-stage aim of this study. The clinical and patient-reported outcomes (Section "Clinical Outcomes at 6 Months" and "Patient-Reported Outcomes (OHIP-14)") — union, occlusal stability, mouth opening, lateral excursion, neurosensory status, OHIP-14, and patient-reported satisfaction — are secondary and descriptive; the study was not powered to assess clinical endpoints.

### Reporting and participant flow

This is a single-arm, non-randomised interventional feasibility study, for which no fully matching EQUATOR reporting checklist exists. Because randomisation, allocation concealment, and between-group comparison do not apply, the randomised-trial reporting guidelines (CONSORT 2010 and its pilot and feasibility extension [25]) were not adopted as a compliance standard. For transparency, participant flow is nonetheless reported in a summary table (Table 1) adapted from the CONSORT flow format, with randomisation- and allocation-related items omitted as not applicable.

**Table 1:** Summary of participant flow

Variable	Number
Assessed for eligibility (screened)	9
Excluded (did not meet criteria)	3
Enrolled	6
Received the intervention	6
Completed 4-week postoperative CT	6
Completed 12-week OHIP-14	6
Completed 6-month clinical follow-up	6
Analysed (primary outcome)	6
Withdrawals / lost to follow-up	0

## Results

### Participant flow summary

All six enrolled patients received the intervention and completed the 4-week postoperative CT scan, the 12-week OHIP-14 assessment, and the 6-month clinical follow-up. No withdrawals or losses to follow-up occurred. Table 1 summarises the flow.

### Sample characteristics

Six patients (four males, two females), mean age 25.3 ± 6.8 years. Fracture locations: left body (*n* = 3), right body (*n* = 3). Mean time from injury to surgery was 4.2 days (range 2–7 days). All procedures completed without intraoperative complications.

### Plan–execution fidelity — primary composite (10 measurements)

#### Patient-level MAE

Range 0.25–0.81 mm (mean 0.48 mm, median 0.44 mm, SD 0.21 mm). Across all six cases, ICP registration met the pre-specified acceptance threshold, with a mean achieved RMS surface deviation of 0.27 mm.

#### Measurement-level MAE

60 measurements, MAE = 0.45 mm (SD 0.52 mm, median 0.25 mm, range 0.02–2.58 mm).

#### Intercanine distance (*n* = 6)

MAE = 0.14 mm (SD 0.10 mm). All six measurements were within 0.5 mm of the planned value.

### Exploratory condylar composite (#1, #2)

MAE = 1.15 mm (lateral) and 1.47 mm (medial). Errors > 2.0 mm occurred only in Cases 1–2 (open superior design). Reported here for completeness; not part of the primary fidelity estimand.

### Plate design — descriptive non-comparative pattern

Plate design, indication, and chronological order are structurally confounded. Patient-level MAE ranged from 0.25 mm (closed design, later case) to 0.81 mm (open superior design, early case). No comparative interpretation is permissible. Detailed chronological case data are in Table 2, which reports the all-12-measurement (condylar-inclusive) error for transparency; the primary 10-measurement composite is summarised in Section "Plan–Execution Fidelity — Primary Composite (10 measurements)".

**Table 2:** Chronological case data (confounded — no valid inference on plate design)

Case	Age/Sex	Fracture side	Plate design	Mental nerve involved?	Mean absolute error (mm) — all 12 measurements (incl. condylar #1–2)	Max absolute error (mm) — all 12	Key observation
1	24/M	Left	Open superior	Yes	1.25	4.55	Large posterior condylar deviation
2	29/F	Left	Open superior	Yes	0.72	3.20	Moderate posterior deviation
3	18/M	Right	Closed	No	0.47	1.14	All errors < 2.0 mm
4	17/M	Right	Closed	No	0.40	1.58	All errors < 2.0 mm
5	31/F	Left	Closed	No	0.39	0.95	All errors < 2.0 mm
6	33/M	Right	Closed	No	0.30	0.88	All errors < 2.0 mm

Values in the two error columns are the mean and maximum absolute error across all 12 measurements (11 CT-based plus the intraoral-scan-based intercanine distance, #8), including the two exploratory condylar metrics (#1 and #2); they are shown for completeness and are not the primary endpoint. The pre-specified primary endpoint — the patient-level MAE of the 10-measurement composite with condylar metrics excluded — is reported in Section "Plan–Execution Fidelity — Primary Composite (10 measurements)" (mean 0.48 mm, median 0.44 mm, range 0.25–0.81 mm, SD 0.21 mm). Because condylar deviations were largest in the two earliest (open-superior) cases, the all-12 error values for Cases 1 and 2 exceed their corresponding 10-measurement composite values. All values are descriptive; no confidence intervals or inferential statistics are reported

### Clinical outcomes at 6 months

Fracture union was assessed clinically at follow-up visits by absence of mobility across the fracture site, restoration of function, and lack of symptoms requiring further intervention. No routine CT imaging was performed after the 4-week postoperative scan. At 6 months, mean maximum incisal opening was 42.3 mm (range 38–48 mm); mean lateral excursion was 8.2 mm (range 7–10 mm). The following adverse events were recorded (the study was not powered to evaluate complication rates; these observations are reported descriptively only):

**Table:** 

Outcome	Number of patients (*n* = 6)
Infection	0
Plate exposure	0
Non-union	0
Malunion requiring reoperation	0
Hardware fracture	0
Persistent malocclusion	0
Persistent neurosensory disturbance	0
Reoperation (any cause)	0

Thus, no infection, plate exposure, non-union, malocclusion requiring adjustment, hardware fracture, or reoperation was observed during the 6-month follow-up. No patient reported persistent neurosensory deficits. At the final follow-up visit, all six patients expressed satisfaction with their facial appearance and with the procedure; this was recorded as a descriptive, qualitative report and was not captured with a validated satisfaction instrument. Because no patient experienced persistent malocclusion during the 6-month follow-up, a correlation analysis between occlusal disturbances and surface deviation could not be performed.

### Patient-reported outcomes (OHIP-14)

OHIP-14 total scores improved from 48.2 (SD 5.6) preoperatively to 35.8 (SD 6.2) at 1 week, 19.5 (SD 5.1) at 4 weeks, and 5.2 (SD 4.1) at 12 weeks. Table 3 presents individual patient OHIP-14 domain scores over time. These are descriptive recovery data and cannot be attributed specifically to the digital workflow.

**Table 3:** OHIP-14 domain scores over time by patient

Patient	Time point	Functional limitation	Physical pain	Psychological discomfort	Physical disability	Psychological disability	Social disability	Handicap
1	Pre	4	4	3	3	4	3	2
	1 Week	3	4	2	2	3	3	1
	4 Weeks	1	2	1	1	1	2	1
	12 Weeks	0	1	0	0	1	1	0
2	Pre	4	4	3	4	3	3	2
	1 Week	3	3	2	3	2	2	1
	4 Weeks	2	2	1	1	1	1	1
	12 Weeks	0	0	0	0	0	1	0
3	Pre	4	4	3	4	3	3	2
	1 Week	3	3	2	2	2	3	1
	4 Weeks	1	1	1	1	1	2	1
	12 Weeks	0	0	0	0	0	1	0
4	Pre	4	3	4	4	3	4	2
	1 Week	3	3	3	3	2	3	1
	4 Weeks	2	2	2	2	2	2	1
	12 Weeks	1	1	1	1	1	1	0
5	Pre	4	3	3	3	3	3	1
	1 Week	2	3	2	2	2	2	1
	4 Weeks	1	1	1	1	1	1	0
	12 Weeks	0	0	0	0	0	0	0
6	Pre	4	4	4	4	3	4	3
	1 Week	3	3	2	3	2	3	1
	4 Weeks	1	2	1	1	1	1	1
	12 Weeks	0	1	0	0	0	1	0

Each cell is the domain score (0–4 scale per item summed within domain). Higher scores indicate greater impact. Values are descriptive

## Discussion

### Interpretation of the primary outcome

This pilot study assessed plan–execution fidelity — the geometric agreement between the virtually planned and postoperative mandibular morphology after image registration — in a fully digital workflow using custom-made plates. The observed patient-level MAE was 0.48 mm (range 0.25–0.81 mm). Below we discuss what this metric represents, what the study contributes, and its limitations in the context of a small exploratory cohort.

The MAE quantifies within-workflow congruence after ICP registration on the contralateral ramus/condyle. Because registration is performed on the contralateral ramus and condyle, measurements located on or adjacent to that reference segment are constrained toward near-zero deviation by construction; the composite MAE therefore partly reflects registration quality rather than execution error alone. This is underscored by the proximity of the achieved registration error (mean RMS surface deviation 0.27 mm) to the reported composite MAE (0.45–0.48 mm), which indicates that the measured deviation lies only modestly above the registration noise floor and should be interpreted accordingly; consistent with the descriptive framing in Section "Statistical Approach and Outcome Hierarchy", the reported figures are therefore presented as approximate within-sample estimates rather than as precise measures of accuracy. Because the metric is registration dependent and lacks an independent anatomical reference, it should be understood as a measure of workflow reproducibility — how faithfully the executed result reproduces the digital plan — rather than as a measure of surgical or anatomical accuracy. The clinical value of this study therefore lies not in establishing that a particular deviation is clinically optimal, but in providing a transparent, reproducible method for reporting plan-to-execution transfer in digital trauma workflows. No validated threshold currently links such fidelity measurements to clinical outcomes (e.g., occlusal stability, union, or patient-reported recovery) in mandibular fracture surgery; establishing such thresholds is a necessary subject for future, adequately powered comparative work. Accordingly, the submillimetre values reported here should be read as feasibility and consistency data, not as evidence of clinical superiority.

Critically, the geometric fidelity metric was not the means by which we judged whether the digital plan had been clinically achieved. Because this single-arm study had neither a contralateral anatomical ground truth nor a parallel control group, clinical achievement of the planned reduction was verified against the two accepted clinical reference standards for mandibular fracture treatment. Restoration of a stable, functional occlusion is the principal functional endpoint and the practical reference for the adequacy of mandibular fracture reduction [13], and re-establishment of mandibular bony continuity with uneventful union is the accepted criterion of successful healing [15]. In this cohort, all patients achieved a stable, reproducible occlusion without the need for postoperative adjustment, together with clinical and radiographic re-establishment of mandibular continuity and uneventful union (Section "Clinical Outcomes at 6 Months"), indicating that the fully digital sequence — virtual planning, occlusal stent, surgical guide, and patient-specific plate — was clinically achieved. Descriptively, the submillimetre plan–execution fidelity observed here co-occurred with these uniformly favourable clinical findings — restored occlusion, uneventful union, OHIP-14 recovery, and an absence of complications (Section "Clinical Outcomes at 6 Months" and "Patient-Reported Outcomes (OHIP-14)"); this co-occurrence cannot establish that any particular deviation magnitude causes a given clinical outcome, but it indicates that faithful transfer of the plan was achievable alongside satisfactory early clinical results in this cohort. We therefore validated the workflow against these clinical endpoints rather than against the contralateral (mirrored) side or a separate control group: contralateral mirroring is constrained by the known prevalence of mandibular asymmetry [26, 27] (Section "Limitations"), and a single-arm pilot has no control group, whereas occlusion and bony continuity are the criteria by which the success of fracture reduction is routinely judged in clinical practice [13, 15]. Within this framework, the geometric MAE is complementary: it quantifies the faithfulness of plan-to-execution transfer, while occlusion and bony continuity establish that the planned reduction was clinically realised.

### Comparison with previous literature

The observed mean MAE of 0.48 mm (range 0.25–0.81 mm) falls within the range of previously reported patient-specific implant accuracy studies [28]. For mandibular reconstruction, Zweifel et al. (2020) reported a mean deviation of 0.65 mm (range 0.3–1.2 mm) for patient-specific plates in angle fractures [9]. He et al. (2021) described an average error of 0.71 mm (SD 0.28 mm) for a digital workflow in mandibular fractures [29]. In orthognathic surgery, transfer accuracy of virtual planning to postoperative result is typically reported between 0.5 mm and 1.0 mm for linear measurements [8]. Our findings are numerically comparable to these studies, although direct comparisons are limited by differences in registration protocols, anatomical landmarks, case mix, and outcome definitions.

### What this pilot contributes

Despite the small sample size, this study provides a transparent template for reporting plan–execution fidelity in digital trauma workflows. Methodological strengths include: pre-specified exclusion of condylar metrics (due to high sensitivity to registration instability), descriptive patient-level MAE accounting for clustering, deliberate restriction to a single, homogeneous fracture type to limit between-patient variability arising from differing fracture patterns, and explicit acknowledgment of confounding in plate design comparisons. The workflow was successfully implemented in six consecutive patients, with no intraoperative complications or revision surgeries.

### Observations on plate design and learning curve

Plate design, indication, and chronology are fully confounded; therefore, no causal or comparative inference is possible. The two earliest cases used the open-superior design and showed the largest deviations — a pattern equally attributable to an early learning-curve effect, to the anatomical indication (mental-nerve involvement), or to the structural difference between the open and closed constructs [30], none of which can be separated in six patients. We therefore draw no design-related conclusion and offer the observation only as a hypothesis for future controlled evaluation. The absence of hardware failure is consistent with adequate construct stability, but no causal link to plate design can be drawn.

### Patient-reported outcomes

The OHIP-14 improvement from 48 to 5 at 12 weeks is numerically comparable to published postoperative ranges in mandibular trauma cohorts [31, 32]. These data are descriptive of recovery after surgery and cannot be attributed specifically to the digital workflow, particularly in the absence of a control group.

### Cost considerations

Detailed cost data were not prospectively collected; therefore, no monetary estimates are provided. However, the described workflow requires additional resources compared with conventional mandibular fracture fixation — including virtual surgical planning, computer-aided design/computer-aided manufacturing (CAD/CAM) engineering, additive manufacturing of guides and stents, production of patient-specific implants, and postoperative imaging. These expenditures must ultimately be weighed against potential benefits such as elimination of intraoperative plate bending, improved transfer of the virtual plan, and greater procedural standardisation. Formal economic evaluation was beyond the scope of this pilot study; consequently, the present study cannot determine whether the observed geometric fidelity justifies the additional manufacturing and imaging costs.

### Limitations

The primary limitation is the very small sample size (*n* = 6), which makes all estimates descriptive and not generalisable. Additional limitations include:

- No external anatomical validation; the MAE is registration dependent and partially circular.
- An unvalidated assumption of contralateral symmetry, given the known prevalence of mandibular asymmetry.
- Absence of a control group, precluding any claim of superiority, equivalence, or clinical advantage. This was a deliberate design choice rather than an oversight: the question addressed here is whether a principled virtual plan can be transferred to the patient reliably, which is logically prior to — and a precondition for — any comparative trial. Under staged frameworks for surgical innovation [14], such feasibility and reproducibility must be established before randomised comparison is warranted; a control arm is therefore the appropriate design for the next stage, not this one.
- Retrospective trial registration (though registration was completed before data analysis and no outcomes were changed).
- Structural confounding of the plate design comparison.
- Potential instability of ICC estimates derived from only six patients, and observer averaging that smooths true measurement variability.
- Short follow-up (6 months) and use of a general quality-of-life instrument (OHIP-14) that may not capture all trauma-specific dimensions, as no validated mandibular-fracture-specific patient-reported outcome measure was available in Arabic.
- Clinical assessment was limited to union, occlusal stability, neurosensory status, mouth opening, lateral excursion, and patients’ self-reported satisfaction with appearance and treatment; formal aesthetic outcome measures, masticatory-performance testing, validated patient-satisfaction instruments, and long-term implant performance were not systematically evaluated and remain objectives for future study.
- Workflow efficiency and treatment delay attributable to PSI fabrication were not systematically evaluated (though all surgeries occurred within 7 days), and detailed cost data were not collected (see Section "Cost Considerations").
- Because postoperative assessment relied on CT imaging acquired four weeks after surgery, minor biological remodelling during early healing may have contributed to measured deviations.
- The protocol required an additional postoperative CT scan for fidelity assessment, entailing radiation exposure beyond standard follow-up used in some centres.
- All procedures were performed by a single highly experienced surgeon (> 28 years); the observed fidelity may not be reproducible among surgeons with different experience levels.

### Conclusions

In this pilot cohort, a fully digital workflow using custom-made plates for mandibular fracture repair was associated with submillimetre plan–execution deviation (mean 0.48 mm) within the registration framework used in this study. The workflow was technically feasible and showed close correspondence between the virtual plan and postoperative morphology. Whether this degree of digital congruence translates into clinically meaningful advantages over conventional fixation remains to be established in adequately powered comparative studies. Observations regarding plate design remain hypothesis generating only. These preliminary findings provide a foundation for future controlled studies aimed at evaluating clinical relevance, workflow efficiency, economic impact, and long-term outcomes in larger patient cohorts.

## Supplementary Information

Supplementary Material 1.

## Data Availability

The data supporting this study are available from the corresponding author upon reasonable request. Supplementary Table S1 (measurement definitions) is provided as a supplementary file with this submission.
