# Supplementary material for: Assessment of plan–execution fidelity in a fully digital workflow using custom-made plates for mandibular fracture repair: a prospective pilot study
Source: BMC Oral Health. 2026 Jul 22;26:1331. doi: 10.1186/s12903-026-09349-5 (PMC13397634; doi:10.1186/s12903-026-09349-5)
Supplement: Supplementary file 1 — Supplementary Material 1. [file 12903_2026_9349_MOESM1_ESM.docx]

**Supplementary Table S1. Detailed landmark definitions with 3D placement criteria**

| **#** | **Measurement** | **Abbr.** | **Landmark definition (3D)** | **3D placement criteria** | **Primary/Exploratory** |
| --- | --- | --- | --- | --- | --- |
| 1 | Lateral Intercondylar Length | LIL | Most lateral point on the lateral pole of each condyle | Axial view: point at maximum lateral extent. Coronal view: verified at same superior–inferior level as medial point | Exploratory |
| 2 | Medial Intercondylar Length | MIL | Most medial point on the medial pole of each condyle | Axial view: point at maximum medial extent. Excludes osteophytes | Exploratory |
| 3 | Bicoronoid Length | BCL | Tip of the coronoid process (most superior point) | Sagittal view: highest point. Coronal view: verified as not part of condyle | Primary |
| 4 | Bigonial Width | BGW | Most inferior, lateral point on the mandibular angle (gonion) | Intersection of lines tangent to posterior ramus and inferior body (bisected angle method) | Primary |
| 5 | Bimental Width | BMW | Most inferior point on the margin of the mental foramen | Axial view: identified as foramen opening. 3D: point at 6 o'clock position relative to foramen center | Primary |
| 6 | Maximum Mandibular Length — Right | MML R | Distance from pogonion to perpendicular line tangent to posterior point of right condyle | Sagittal view: pogonion (most anterior midline point) to line perpendicular to Frankfort horizontal at posterior condyle | Primary |
| 7 | Maximum Mandibular Length — Left | MML L | Distance from pogonion to perpendicular line tangent to posterior point of left condyle | Same as above | Primary |
| 8 | Intercanine Length | ICL | Tip of the canine cusp (right and left) | Intraoral scan: highest cusp point relative to occlusal plane. Requires intact canine | Primary |
| 9 | Maximum Ramus Height — Right | MRH R | Distance from highest point on condylar head to gonion (right) | Sagittal view: straight line, not following bone curvature | Primary |
| 10 | Maximum Ramus Height — Left | MRH L | Distance from highest point on condylar head to gonion (left) | Same as above | Primary |
| 11 | Mandibular Angle — Right | MA R | Angle between inferior border of body and posterior border of ramus (right) | 3D: measured on lateral projection. Defined by lines tangent to posterior ramus and inferior body | Primary |
| 12 | Mandibular Angle — Left | MA L | Angle between inferior border of body and posterior border of ramus (left) | Same as above | Primary |

*Notes for measurement reproducibility:*

*• All landmarks placed at 3× magnification in Mimics Medical 24.0.*

*• For bilateral measurements (#3–12), right and left sides measured separately and reported as individual readings.*

*• In cases with a fracture line through a landmark region, the contralateral unaffected side was used as reference for the planned model; postoperative measurements used both sides with verification of healing.*

*• Intra-observer reliability: ICC(2,1) = 0.94 (Shrout and Fleiss [21]).*

*• Inter-observer reliability: ICC(2,k) = 0.92 (average of three raters).*

*• Primary interpretation excludes exploratory condylar metrics (#1, #2).*
